# Spatial lipidomics in gynecologic malignancies: molecular identification, cellular attribution, and mechanistic validation

**DOI:** 10.3389/fmolb.2026.1957466

**Published:** 2026-10-13

**Authors:** Jingyan Zhao, Wei Ma

**Affiliations:** Medical Department, Northwest Minzu University, Lanzhou, Gansu Province, China

**Keywords:** ferroptosis, gynecologic malignancies, lipid metabolism, mass spectrometry imaging, metabolomics, spatial lipidomics, tumor microenvironment

## Abstract

Spatial lipidomics can place lipid identities within histopathologic compartments, but ion maps are frequently interpreted beyond what their analytical and biological validation can support. Here, we critically synthesize direct spatial lipid evidence in ovarian, endometrial, and cervical cancers and use spatial metabolomics, mass spectrometry imaging, spatial transcriptomics or proteomics, and non-spatial lipid biology only as explicitly graded support. We organize inference as an eight-gate chain: pathology-defined sampling, analytical quality control, structural identification, cellular attribution, metabolic flux, perturbation and rescue, patient-level validation, and prospective clinical utility. Within this framework, lipid uptake, *de novo* synthesis, desaturation, fatty-acid oxidation, membrane remodeling, prostaglandin E2 signaling, and ferroptosis are interpreted as mechanistic programs downstream of spatially anchored observations. Disease ecology determines which programs are plausible: omental adipocyte-tumor exchange dominates ovarian cancer, obesity-estrogen-insulin and PI3K signaling frame endometrial cancer, and persistent high-risk human papillomavirus infection shapes membrane and inflammatory remodeling in cervical cancer. Evidence remains highly asymmetric. Ovarian cancer has the broadest spatial literature; endometrial evidence relies principally on one 64-patient DESI-MSI cohort; and cervical evidence is limited to very small phospholipid and oleate studies. Spatial lipidomics is therefore ready to localize disease-specific niches, prioritize causal experiments, and define testable patient subsets, but not yet to support routine screening, diagnosis, treatment selection, or surveillance. Closing the gap requires orthogonal structural confirmation, cell-resolved source assignment, isotope tracing, functional rescue, and locked multicenter validation.

## Introduction

Ovarian, endometrial, and cervical cancers are not one disease distinguished only by anatomic site. They have different origins, dominant drivers, and clinical trajectories. High-grade serous ovarian cancer is commonly associated with precursor lesions in the fimbrial fallopian tube and is often detected after peritoneal-omental dissemination. Endometrial cancer burden is closely related to obesity, metabolic dysfunction, and molecular subtype. Cervical cancer is driven by persistent high-risk human papillomavirus (HPV) infection and is substantially preventable through vaccination and screening (American Cancer Society, 2026a; Berek et al., 2021; Bray et al., 2024; Falcaro et al., 2021; Lei et al., 2020; Ronco et al., 2014; World Cancer Research Fund, 2026a; World Cancer Research Fund, 2026b; World Cancer Research Fund, 2026c; World Health Organization, 2021; World Health Organization, 2026). This Review examines how lipid remodeling is embedded in these disease-specific trajectories and whether spatial lipid information can complement pathology, molecular classification, and clinical decision-making.

Etiology and lipid metabolism are connected through an adaptive interaction between cell-intrinsic alterations and local ecological pressures. Ovarian cancer cells entering ascites and the lipid-rich omentum must withstand detachment, nutrient fluctuations, and chemotherapy. They can mobilize adipocyte-derived lipids, *de novo* synthesis, lipid-droplet storage, and fatty-acid oxidation (FAO) (Mukherjee et al., 2023; Nieman et al., 2011; Zhao et al., 2019). In endometrial cancer, obesity can increase postmenopausal estrogen exposure through peripheral aromatization and is accompanied by insulin resistance and chronic low-grade inflammation. These signals converge with frequent phosphoinositide 3-kinase-protein kinase B (PI3K-AKT) pathway abnormalities and sterol regulatory element-binding protein 1 (SREBP1)-driven lipogenesis, converting host metabolic conditions into membrane production and growth signaling (American Cancer Society, 2026b; Aune et al., 2015; Li et al., 2012; Wang et al., 2020; Zhang et al., 2017). In cervical epithelium, HPV E5, E6, and E7 oncoproteins disrupt cell-cycle and genomic control. They also alter glycerophospholipids, sphingolipids, oxidized lipids, and cyclooxygenase-prostaglandin E2 (PGE2) signaling (Crosbie et al., 2013; Gore et al., 2025; Oh et al., 2009; Spurgeon et al., 2026; Subbaramaiah and Dannenberg, 2007). The three cancers can therefore share lipid functions without sharing the same upstream cause, dominant compartment, or clinical consequence.

These different clinical courses define distinct spatial questions. Ovarian cancer requires comparison of primary tumors, omental metastases, ascitic aggregates, and post-treatment residual disease to identify metastatic and resistant compartments. Endometrial cancer requires lipid mapping across malignant glands, stroma, vessels, and the myometrial invasive front, followed by testing for added value beyond molecular classification. Cervical cancer is better studied across lesion-progression interfaces, invasive fronts, lymph-node metastases, and residual regions after chemoradiotherapy (Cibula et al., 2023; Concin et al., 2025; González-Martín et al., 2023). Each setting poses the same testable questions: which lipids change, where they change, which cells and metabolic processes produce the change, and whether the spatial state is associated with a defined clinical outcome.

Bulk and spatial lipidomics share mass spectrometry, lipid annotation, tandem mass spectrometric confirmation, and statistical modeling. Their critical difference is whether tissue coordinates are retained. Tissue homogenization enables deeper coverage and relatively stable quantification, but produces a weighted average of tumor, stroma, vessels, adipose tissue, and necrosis. Spatial lipidomics instead acquires mass spectra across a tissue section and registers ion maps with pathology. It can therefore localize candidate signals to malignant epithelium, stroma, adipose tissue, vessels, necrosis, or invasive fronts. This localization gain reveals heterogeneity concealed by tissue averages, but colocalization alone cannot identify a producing cell or distinguish synthesis, uptake, storage, and degradation (Dória et al., 2016; Paraskevaidi et al., 2025; Xu et al., 2025; Zhang et al., 2026).

Direct spatial lipid measurement in tumor tissue is the evidentiary core of this Review. Spatial metabolomics, general MSI, spatial transcriptomics or proteomics, and non-spatial lipid mechanisms provide process-, source-, or function-level support but are not treated as interchangeable evidence. Recent reviews have synthesized pan-cancer metabolic rewiring and phosphatidylcholine-ferroptosis crosstalk in the tumor microenvironment (Kaushik et al., 2026; Vani Raju et al., 2026); neither ranks direct spatial lipid evidence across gynecologic malignancies or tests how far each ion map supports source, flux, function, and clinical inference. We therefore pursue three aims. First, we define an eight-gate framework from pathology-defined sampling to prospective utility. Second, we compare how uptake, *de novo* synthesis, desaturation, FAO, membrane remodeling, PGE2 signaling, and ferroptosis are embedded in the etiologic ecology of each cancer. Third, we distinguish direct evidence from testable hypotheses and currently unsupported clinical uses. Because evidence depth differs markedly across diseases, the synthesis remains intentionally asymmetric (Dória et al., 2016; Leung et al., 2024; Mondal et al., 2019; Paraskevaidi et al., 2025; Pietkiewicz et al., 2022; Xu et al., 2025; Zhang et al., 2026).

Figure 1 summarizes the evidence hierarchy and the deliberately asymmetric comparison across ovarian, endometrial, and cervical cancers.

**FIGURE 1:**
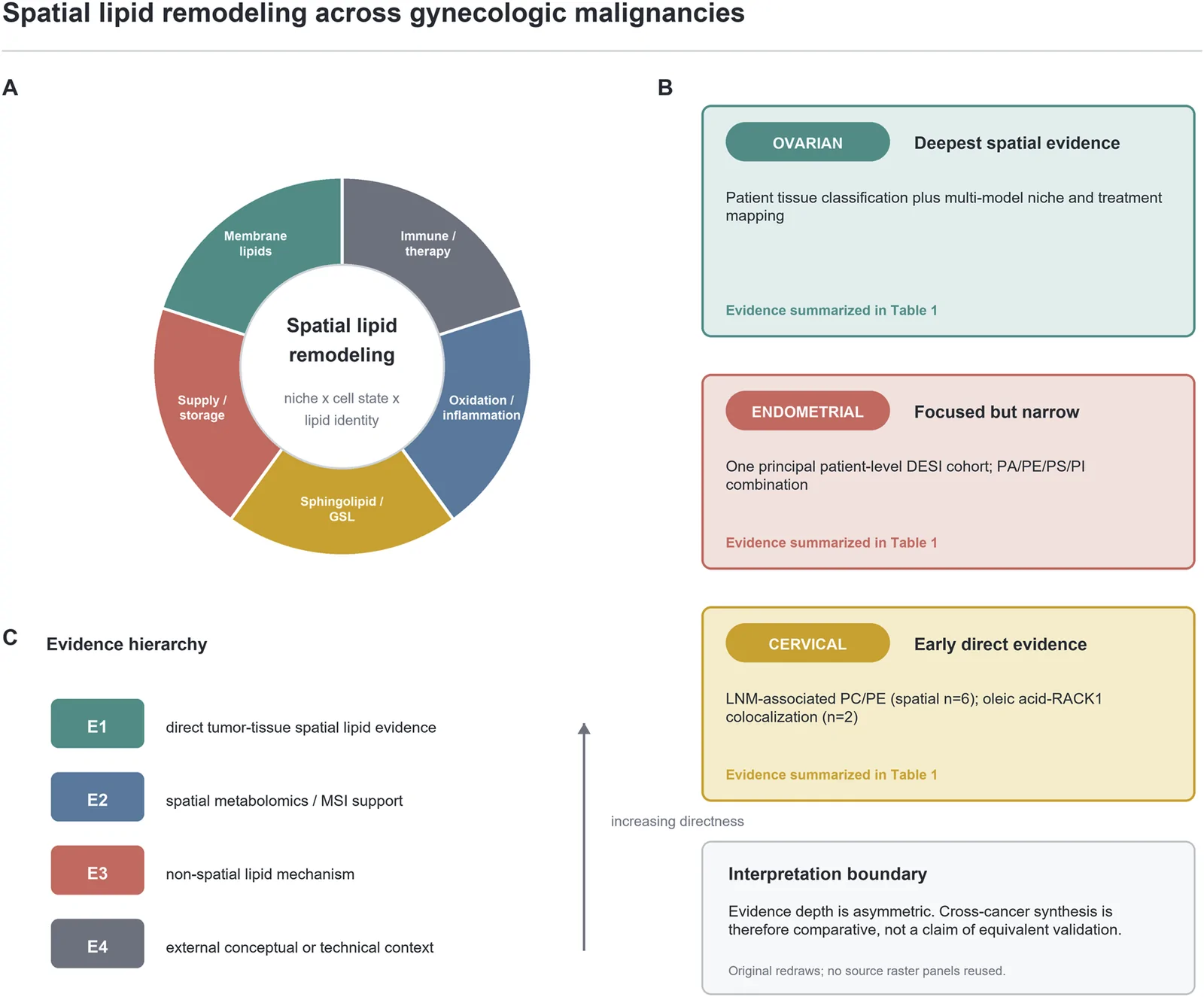
Overview of spatial lipid remodeling in gynecologic malignancies. The figure contrasts the omental lipid ecology of ovarian cancer, the obesity-hormone-insulin context of endometrial cancer, and HPV-associated epithelial remodeling in cervical cancer. Evidence is stratified as direct spatial lipid evidence, supporting spatial evidence, or non-spatial mechanistic evidence. Key mechanistic sources are cited in references (Dória et al., 2016; Li et al., 2012; Mukherjee et al., 2023; Nieman et al., 2011; Paraskevaidi et al., 2025; Razghonova et al., 2025; Spurgeon et al., 2026; Xu et al., 2025; Zhang et al., 2026; Zhao et al., 2019). **(A)** Conceptual domains of spatial lipid remodeling. **(B)** Disease-specific evidence depth in ovarian, endometrial, and cervical cancers, together with the interpretation boundary. **(C)** Evidence hierarchy from E1 direct tumor-tissue spatial lipid evidence to E4 external conceptual or technical context.

### Spatial lipidomics as an evidentiary system: from ion map to mechanism

#### Review scope and evidence hierarchy

In this Review, direct spatial lipid evidence (E1) denotes a lipid molecule or lipid-annotated ion measured in tumor tissue while tissue coordinates are retained. Spatial metabolite maps that are not lipid-specific and spatial transcriptomic or proteomic proxies provide supporting spatial evidence (E2); non-spatial lipid mechanisms provide functional context (E3); and external technical or clinical sources provide contextual evidence (E4). Evidence is interpreted only to the highest level actually supported: regional localization, cellular source, metabolic flux, causal function, patient-level validity, or clinical utility. Figure 1 summarizes this hierarchy and the unequal evidence depth across the three diseases.

#### Analytical control and molecular identification

Spatial lipidomics is not the name of a single instrument. It is a strategy for detecting, identifying, and interpreting lipids while preserving their tissue locations. A typical workflow comprises eight steps. The first four are to prespecify clinical and sampling questions, control analytical quality, acquire MSI data, and establish molecular identity. The next three assign cellular origin, resolve metabolic flux by stable-isotope tracing, and test function through perturbation and rescue. The final step evaluates added clinical value in an independent patient cohort. The first four steps establish measurement credibility, the next three address mechanism, and the last establishes clinical value. Evidence at one level cannot substitute automatically for the next (Dória et al., 2016; Paraskevaidi et al., 2025; Pietkiewicz et al., 2022; Xu et al., 2025; Zhang et al., 2026).

Figure 2 summarizes the transition from controlled tissue sampling and lipid identification to cellular attribution, causality, and patient-level validation.

**FIGURE 2:**
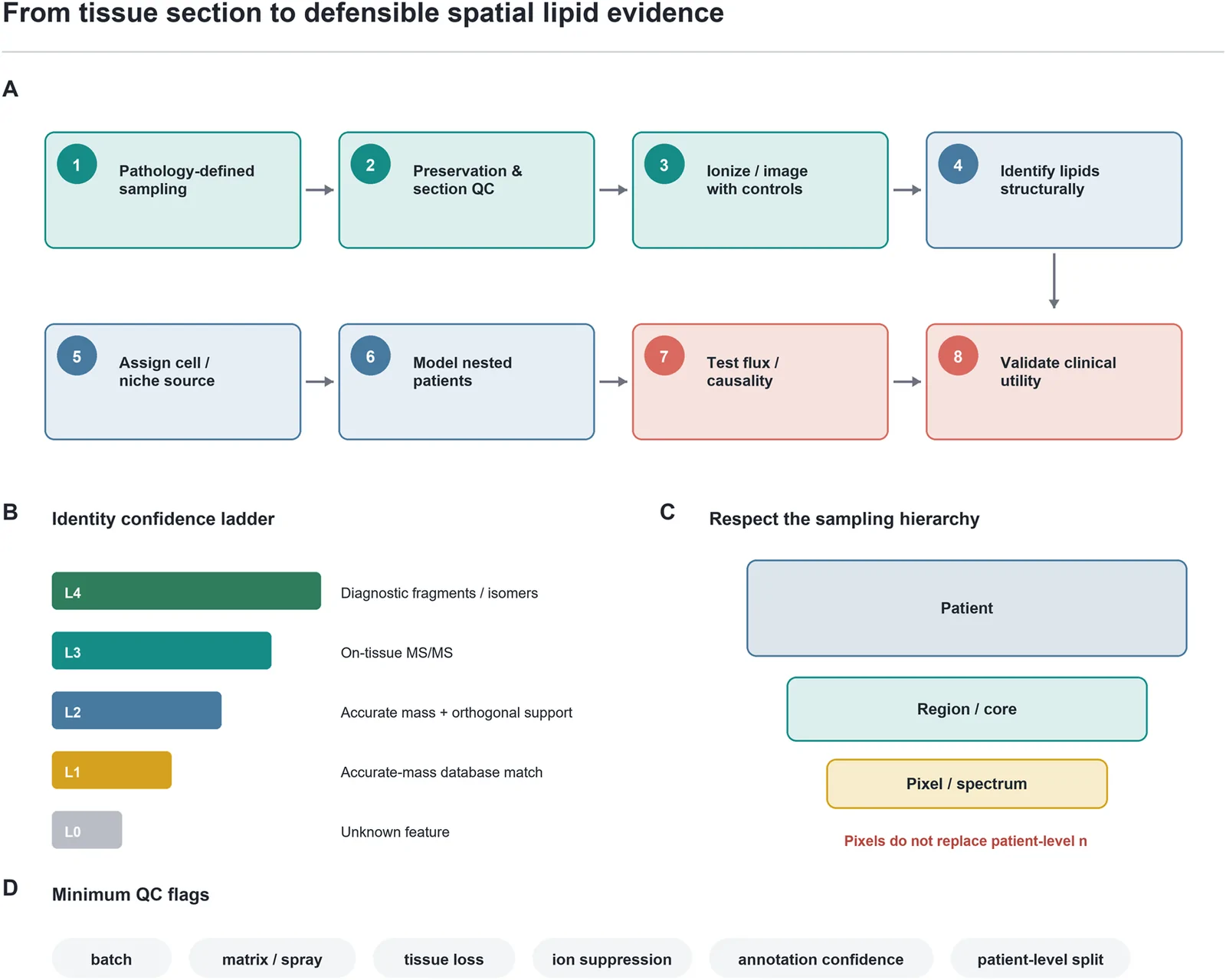
A tiered framework for defensible spatial lipid evidence. Direct lipid measurement, supporting spatial metabolism, spatial multi-omic proxies, and non-spatial mechanisms permit different levels of inference. Regional localization does not automatically establish cellular origin, metabolic flux, causality, or clinical utility (Dória et al., 2016; Paraskevaidi et al., 2025; Pietkiewicz et al., 2022; Xu et al., 2025; Zhang et al., 2026). **(A)** Eight-step workflow from pathology-defined sampling to clinical validation. **(B)** Lipid-identification confidence ladder from L0 to L4. **(C)** Patient-region/core-pixel/spectrum sampling hierarchy. **(D)** Minimum quality-control flags.

The clinical question must first be translated into a sampling design. For omental metastasis, for example, the tumor-adipose interface, tumor center, and distant adipose tissue should be defined before imaging rather than selected after observing the strongest differences. Pre-analytical control encompasses every step between tissue removal and analysis, including ischemic time, freezing or fixation, storage, section thickness, slide type, washing, and matrix deposition. Lipids can oxidize, degrade, or migrate within sections, creating artifactual regional signals. Studies should therefore capture complete metadata, randomize samples, include process controls and replicate sections, and use the patient rather than the pixel as the independent statistical unit (Condina et al., 2019; Pietkiewicz et al., 2022; Schwamborn, 2017; Thomas and Chaurand, 2014).

MSI acquires a mass spectrum at successive tissue coordinates and reconstructs each mass-to-charge ratio (m/z) as an ion-distribution map. Accurate mass or database matching usually provides only a candidate annotation. *In situ* tandem mass spectrometry (MS/MS), orthogonal liquid chromatography-tandem mass spectrometry (LC-MS/MS), ion mobility, and authentic standards progressively strengthen class- or species-level identification. Double-bond position and stereochemistry require structure-sensitive methods. Reports should state mass error, fragment coverage, identification level, and unresolved structural features. Relative ion intensity should not be presented as absolute concentration (Condina et al., 2019; Nguyen et al., 2024; Xia et al., 2025).

#### Cellular attribution, metabolic flux, and functional validation

Ion maps should next be registered with hematoxylin-eosin or immunopathology images to define malignant epithelium, stroma, necrosis, vessels, adipose tissue, and invasive fronts. Pathologic colocalization identifies a compartment, not necessarily a producing cell. A spatial pixel commonly contains several cell types, and ion intensity reflects cell composition and ionization efficiency. More robust source attribution combines spatial transcriptomic or proteomic data with laser-capture microdissection, sorted-cell lipidomics, *in situ* labeling, or cell-specific perturbation. Registration error and neighborhood sensitivity should also be assessed (Mukherjee et al., 2023; Wu et al., 2025; Xu et al., 2025).

Stable-isotope-labeled glucose, fatty acids, or other substrates can then distinguish *de novo* synthesis, exogenous uptake, lipid-droplet mobilization, and oxidation. Genetic knockdown or knockout and pharmacologic inhibition can test whether a candidate node is required. Rescue with a substrate or downstream product helps exclude nonspecific effects.

#### Patient-level validation and clinical decision gain

Spatial features must finally be locked and tested at the patient level in independent cohorts. Studies should report discrimination, calibration, confidence intervals, failure rates, and gain over standard pathology or molecular models. Regional enrichment is not flux, model sensitization is not patient benefit, and pixel-level accuracy cannot replace independent patient validation (Ge et al., 2026; Gorman et al., 2024; Ledoux et al., 2026; Lin et al., 2026; Schwamborn, 2017).

#### Mechanistic interpretation: seven lipid programs

The seven core programs can be organized as lipid supply, lipid modification and use, and downstream biological consequence. Exogenous uptake links tumor cells to local adipose and stromal sources. Ovarian cancer can exploit fatty acids released by omental adipocytes. In endometrial cancer, long-chain acyl-CoA synthetase 1 (ACSL1) activates fatty acids for subsequent metabolism. In cervical cancer, fatty-acid translocase CD36 cooperates with transforming growth factor beta (TGF-beta) to promote invasion (Deng et al., 2019; Nieman et al., 2011; Zhou et al., 2024). In parallel, fatty acid synthase (FASN) generates fatty acids *de novo* under transcriptional control by SREBP1 and related programs (Du et al., 2022; Li et al., 2012; Ueda et al., 2010). Uptake and synthesis can compensate for one another, so transporter or enzyme expression alone does not identify the dominant lipid source in a patient tumor.

Desaturation and membrane remodeling form a second layer. Stearoyl-CoA desaturase 1 (SCD1) changes fatty-acid double bonds and thereby affects membrane fluidity, receptor organization, and sensitivity to lipid peroxidation. Remodeling of choline phospholipids, sphingolipids, and glycosphingolipids can further alter adhesion and signaling (Liu et al., 2010; Spurgeon et al., 2026; Tesfay et al., 2019; Trousil et al., 2014). This layer is close to the fatty-acid, phospholipid, and sphingolipid distributions observed by MSI. However, imaging initially provides a regional m/z signal. Without MS/MS, standards, and orthogonal lipidomics, a putative peak cannot be treated as a fully defined molecule or as proof of increased enzymatic flux.

Storage and energy use form a third layer. Lipid droplets sequester triacylglycerols and cholesteryl esters, reducing free-fatty-acid toxicity and releasing substrates during nutrient or treatment stress. Carnitine palmitoyltransferase 1A (CPT1A) controls entry of long-chain fatty acids into mitochondrial oxidation. Inhibition of 6-phosphofructo-2-kinase/fructose-2,6-bisphosphatase 3 can also induce lipophagy and alter chemotherapy sensitivity in gynecologic cancer models (Huang et al., 2021; Iwahashi et al., 2021; Mondal et al., 2019). Increased lipid droplets may therefore indicate a reservoir available to resistant cells, or merely reflect hypoxia, injury, or cell composition. Formation, hydrolysis, and oxidation must be measured together to determine direction.

Lipid signaling and oxidative cell death form the fourth layer. Cyclooxygenase 2 (COX-2) converts arachidonic-acid-derived intermediates into PGE2, which can suppress antigen presentation or effector immunity. Supporting evidence exists in ovarian ascites, phosphatase and tensin homolog (PTEN)-deficient endometrial models, and HPV-associated cervical cancer, but cellular origin and spatial distribution have not been closed into one cross-cancer chain (Brencicova et al., 2017; Pan et al., 2015; Subbaramaiah and Dannenberg, 2007). Ferroptosis is driven by the accumulation of peroxidized polyunsaturated phospholipids and is modulated by RNA-binding motif protein 3, solute carrier family 7 member 11 (SLC7A11), glutathione peroxidase 4 (GPX4), and related defenses (Tesfay et al., 2019; Wang et al., 2023; Wang et al., 2025). The contribution of spatial lipidomics is to locate compartments where oxidizable substrates, iron, and defense programs coexist. Perturbation must then determine whether those regions are therapeutically vulnerable. A peroxide-rich ion map alone does not prove that ferroptosis has occurred.

### Ovarian cancer: tubal origin, omental ecology, and post-treatment adaptation

#### How the disease trajectory shapes lipid demand

The high mortality burden of ovarian cancer is driven largely by high-grade serous ovarian carcinoma (HGSOC). HGSOC is commonly associated with tubal epithelial precursor lesions. Shed cells disseminate through ascites and preferentially colonize lipid-rich sites such as the omentum (American Cancer Society, 2026c; Berek et al., 2021; World Cancer Research Fund, 2026c). This trajectory generates three recurrent pressures. Suspended cells must resist anoikis, omental implants must obtain substrates from adipocytes and stroma, and residual cells must maintain membrane repair, energy production, and antioxidant defense after treatment. Ovarian lipid remodeling is therefore not simply a by-product of proliferation. It is an adaptation shaped by peritoneal dissemination and therapeutic selection.

Ovarian cancer is often diagnosed after peritoneal spread, and screening trials in average-risk populations have not reduced mortality. Cytoreductive surgery and platinum-based chemotherapy can produce an initial response, yet recurrence, platinum resistance, and resistance after maintenance therapy remain major limitations (Berek et al., 2021; Burger et al., 2011; Buys, 2011; González-Martín et al., 2019; Menon et al., 2021; Moore et al., 2018; Moore et al., 2021; Pujade-Lauraine et al., 2014; Ray-Coquard et al., 2019; Us Preventive Services Task Force, 2018). Spatial lipidomics should therefore not be framed as a general-population screening tool. More defensible questions are whether omental metastases contain reproducible lipid-supply compartments, whether residual disease has distinct lipid survival states, and whether those states explain recurrence or response beyond pathology and BRCA or homologous-recombination status.

Poly(ADP-ribose) polymerase (PARP) inhibitors impair repair of single-strand DNA damage and increase replication stress, producing synthetic lethality in BRCA1/2-deficient or homologous recombination-deficient tumors (González-Martín et al., 2019; Moore et al., 2018; Ray-Coquard et al., 2019). Lipid metabolism is not an established direct target of PARP inhibition. Resistant cells may nevertheless increase membrane-lipid synthesis, FAO, or ferroptosis defense to tolerate prolonged replication and oxidative stress. Spatial studies should not replace homologous-recombination testing with a lipid map. Instead, paired pre- and post-treatment specimens can determine whether these adaptive programs concentrate in residual regions and explain resistance heterogeneity that standard stratification does not capture.

#### From omental lipid supply to treatment tolerance

Adipocyte-tumor interaction in the omentum is the most disease-specific ovarian lipid axis. Adipocytes release fatty acids and induce CD36 and fatty acid-binding protein 4 in cancer cells. Imaging, stable-isotope tracing, and functional perturbation further suggest that tumor regions near adipocytes alter phospholipid distributions and redirect glucose carbon toward glycerol-3-phosphate (G3P) and glycerolipid synthesis. These changes support invasion and carboplatin tolerance (Ladanyi et al., 2018; Mukherjee et al., 2020; Mukherjee et al., 2023; Nieman et al., 2011). The chain links spatial proximity, substrate exchange, and phenotype, but its modules have not been closed in the same patient cohort. Current data therefore cannot quantify net lipid flux across human omental metastases.

Figure 3 separates the spatially observed adipocyte gradient from complementary tracing and non-spatial mechanistic support.

**FIGURE 3:**
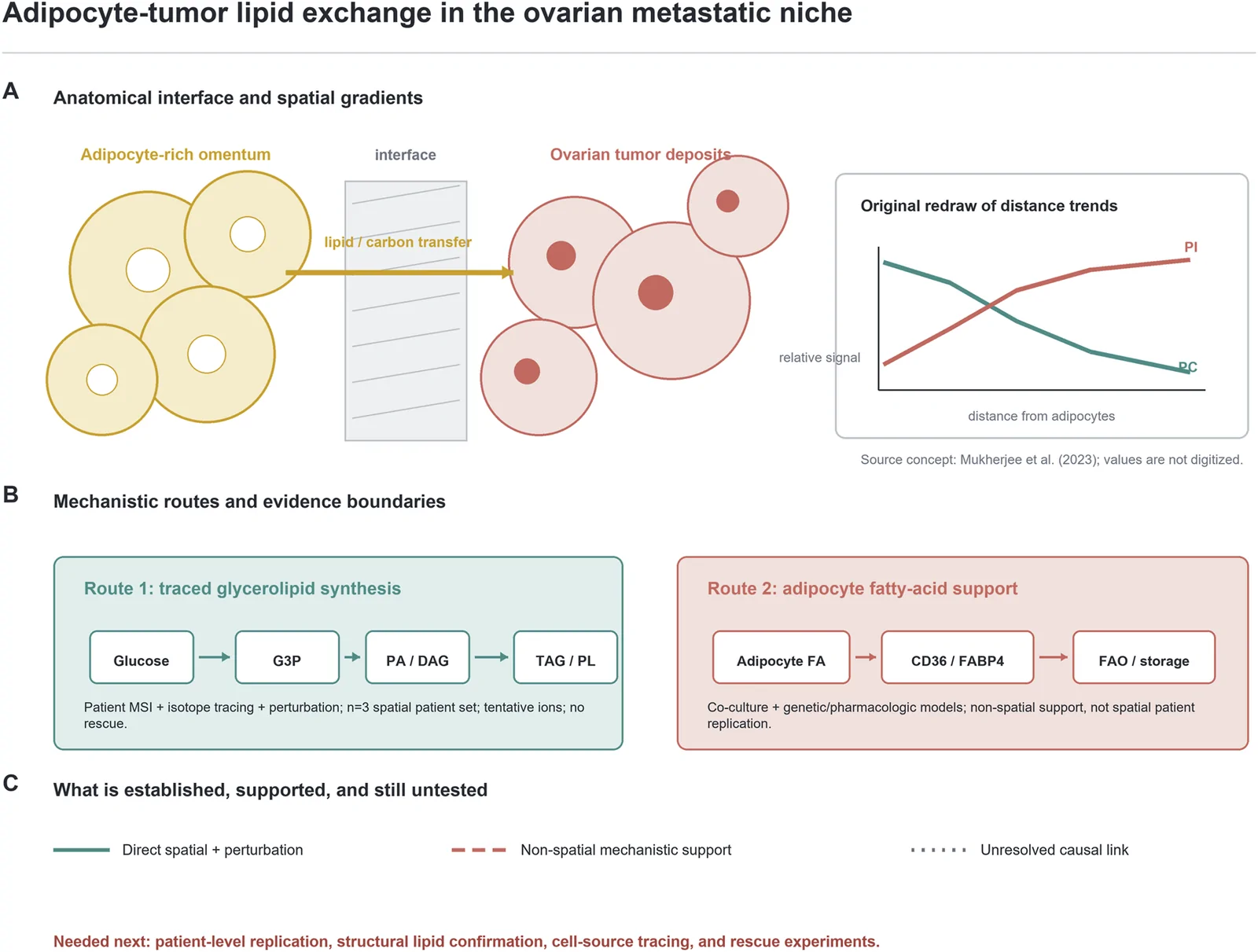
Model of adipocyte-tumor lipid exchange in ovarian cancer. Spatial proximity, substrate tracing, and functional perturbation provide complementary evidence but do not resolve donor, recipient, and net flux within the same patient (Ladanyi et al., 2018; Mukherjee et al., 2020; Mukherjee et al., 2023; Nieman et al., 2011). **(A)** Anatomical adipocyte-tumor interface and spatial gradients, including distance-associated PC and PI trends. **(B)** Traced glycerolipid synthesis and adipocyte fatty-acid support routes. **(C)** Evidence-key categories and unresolved next steps.

Beyond exogenous supply, FASN-mediated synthesis and SCD1/fatty acid desaturase 2 (FADS2)-mediated desaturation can replenish membrane lipids and adjust the ferroptosis threshold. Patient-tissue associations and resistant-cell and xenograft studies link elevated FASN with recurrence and drug tolerance. Nucleus accumbens-associated protein 1 can connect FASN expression to AKT signaling. SCD1 and FADS2 studies further show that unsaturated-lipid balance helps stem-like and ascites-derived ovarian cancer cells resist lipid peroxidation (Bauerschlag et al., 2015; Li et al., 2017; Papaevangelou et al., 2018; Tesfay et al., 2019; Uddin et al., 2011; Ueda et al., 2010; Xuan et al., 2022). Spatial studies should distinguish tumor-intrinsic synthesis from stromal supply and register unsaturated lipids with peroxidizable phospholipids and defense proteins. Drug sensitivity in oleate-supplemented culture cannot define a patient treatment window.

Lipid-droplet storage and FAO provide another buffer against treatment stress. Integrated transcriptomic, proteomic, and metabolomic analysis can nominate CPT1A-associated oxidative dependence. Genetic or pharmacologic perturbation can then test whether resistant cells and animal tumors are resensitized, creating a multi-omic-plus-perturbation chain (Huang et al., 2021; Lin et al., 2025; Nallanthighal et al., 2020; Ma Q. et al., 2023; Yang et al., 2025). Reduced lipid droplet-associated hydrolase can engage adipose triglyceride lipase and CPT1A, whereas argininosuccinate synthase 1 supports anoikis resistance through AMP-activated protein kinase-CPT1A signaling (Deswal et al., 2025; Feng et al., 2025; Iwahashi et al., 2021; Li Y. H. et al., 2026). These findings suggest that residual cells switch among storage, mobilization, and oxidation. Increased droplet abundance may also be a consequence of stress, so droplet formation, hydrolysis, and oxidative flux must be localized together before resistance is inferred.

PGE2 and cholesterol or sphingolipid metabolism may connect metabolic adaptation with immune suppression. PGE2 in ovarian ascites suppresses dendritic cells, and COX-2-associated signaling has been linked to impaired natural killer-cell function (Brencicova et al., 2017; Gómez-Valenzuela et al., 2023). The solute carrier family 7 member 5-ERBB2 axis, sphingosine kinase 1-associated ferroptosis defense, and sterol O-acyltransferase 1-mediated cholesterol esterification have separately been linked to olaparib resistance or CD8 T-cell states (He et al., 2025; Liu et al., 2026; Teng et al., 2025). These pathways have not been demonstrated as one spatial program in patient tissue. Their immediate value is to generate a testable hypothesis that resistant compartments co-localize lipogenesis, ferroptosis defense, and immune exclusion, not to justify a metabolic-immunotherapy combination.

#### Direct spatial evidence and its boundaries

Patient-tissue studies show that MSI can provide localization beyond tissue averages. Desorption electrospray ionization mass spectrometry imaging (DESI-MSI) identified combinations of glycerophospholipid, fatty-acid, and ceramide-related ions that separated normal, borderline, and epithelial ovarian tumors and depicted heterogeneity in serous-tumor aggressiveness. Integration of tissue MSI, *in situ* MS/MS, and transcript data further localized sulfatide enrichment to malignant epithelium (Dória et al., 2016; Liu et al., 2010; Sans et al., 2017). These studies support pathologic-region recognition and candidate-mechanism localization. Most classifications remain internally validated, some ion identities are incomplete, and real-world diagnostic gain over histopathology has not been established. Circulating lysophospholipid associations are non-spatial context and cannot identify a tissue source (Sutphen et al., 2004).

Animal and intervention imaging extend the temporal and functional dimensions. Whole-reproductive-system imaging, spatiotemporal metabolomics, and ultrahigh-resolution MSI in related Dicer/Pten model lineages mapped organ- and stage-dependent glycerophospholipid, sphingolipid, and ether-lipid distributions. Spatial glycosphingolipidomics with genetic perturbation, multimodal imaging after ferroptosis induction, and low-abundance prostaglandin-isomer localization generated plasticity, pharmacodynamic, and inflammatory-lipid hypotheses (Cumin et al., 2022; Gorman et al., 2024; Nguyen et al., 2024; Paine et al., 2016; Sah et al., 2022; Shan et al., 2012; Ma X. et al., 2023). These studies prioritize molecules and regions for human validation. Shared model lineages do not constitute independent patient replication, and animal maps cannot define human screening or response markers. Norepinephrine was the principal analyte in a fallopian tube-ovary crosstalk study; that study illustrates inter-organ imaging but is not direct lipid evidence (Zink et al., 2018).

The near-term priority is multicenter patient-level validation rather than accumulation of unconfirmed regional peaks. Cryogenic three-dimensional imaging, ambient ionization, histology-guided analysis, polarity switching, and online derivatization can improve lipid preservation, coverage, or rapid classification (Kang et al., 2011; Ledoux et al., 2026; Lu et al., 2022; Nazari and Muddiman, 2016; Shon et al., 2025). Their performance must be evaluated with harmonized sampling, pathology dictionaries, structural confirmation, and patient-level statistics. The most informative design would pair primary tumors, omental metastases, and post-treatment residual lesions within BRCA, homologous-recombination, and treatment strata. Locked spatial features should then be tested for added value in recurrence or response models. Current evidence supports niche localization and mechanism discovery, not population screening, routine maintenance selection, or recurrence monitoring.

### Endometrial cancer: membrane remodeling in an obesity-hormone-PI3K context

#### From host risk factors to tumor lipid programs

Endometrial cancer is often detected at a relatively early stage because of abnormal uterine bleeding, but high-risk histologies, deep myometrial invasion, and advanced or recurrent disease still impose substantial treatment burden (American Cancer Society, 2026b; World Cancer Research Fund, 2026b). Its most distinctive etiologic ecology is obesity and metabolic syndrome. Adipose aromatization can increase postmenopausal estrogen exposure, insulin resistance and hyperinsulinemia strengthen growth signaling, and chronic low-grade inflammation alters cytokines and lipid mediators (Aune et al., 2015; Wang et al., 2020). These host factors are not equivalent to intratumoral lipid synthesis. They can nevertheless converge with frequent PI3K-AKT-mechanistic target of rapamycin abnormalities and activate SREBP1/FASN programs that supply fatty acids and membranes. Spatial studies should therefore record adiposity, metabolic state, molecular subtype, and pathologic compartment to separate host context from tumor-cell adaptation.

The 2023 International Federation of Gynecology and Obstetrics staging system and The Cancer Genome Atlas/Proactive Molecular Risk Classifier for Endometrial Cancer framework integrate histopathology with polymerase epsilon (POLE) mutation, mismatch-repair (MMR) deficiency, p53 abnormality, and no specific molecular profile (NSMP) (Berek et al., 2023; Bretová et al., 2025; Jeong et al., 2026; Talhouk et al., 2015; Talhouk et al., 2017; The Cancer Genome Atlas Research Network and Levine, 2013). Fertility-sparing treatment is restricted to carefully selected low-risk disease. High-risk localized and advanced cancers require stratification among surgery, radiotherapy, chemotherapy, immunotherapy, and targeted combinations (Bokhman, 1983; de Boer et al., 2018; Eskander et al., 2023; León-Castillo et al., 2020; Makker et al., 2022; Matei et al., 2019; Mirza et al., 2023; Rodolakis et al., 2023). A spatial lipid feature must therefore address a defined task, such as benign-malignant distinction, myometrial invasion, recurrence, or treatment response. It must add patient-level information beyond current staging and molecular classification rather than be presented as a replacement.

#### Synthesis, membrane remodeling, and lipid fate

The first etiologic link is growth signaling, *de novo* synthesis, and membrane production. FOXO1 regulation of SREBP1-related transcription, FASN expression in patient tissue, and inhibition of lipogenesis in cellular and xenograft models support *de novo* fatty-acid synthesis as a candidate growth program (Li et al., 2012; Razghonova et al., 2025; Tsuji et al., 2004; Zhang et al., 2017). The spatial question is not whether FASN is elevated again, but whether synthesis-active regions lie in malignant glands, myometrial invasive fronts, or defined stromal neighborhoods. Enzyme expression must be registered with directly measured fatty acids and phospholipids. Tracing is then required to distinguish tumor-intrinsic synthesis from lipids supplied by an obese host environment.

The second link is desaturation, membrane remodeling, and signaling adaptation. SCD1 perturbation suppresses endometrial cancer progression. Choline kinase and deacylation pathways alter choline phospholipids, whereas phospholipase D1-mechanistic target of rapamycin complex 2 signaling and patient-tissue sphingolipid changes separately implicate phosphatidic-acid and sphingolipid remodeling (Knapp et al., 2010; Li et al., 2018; Shen et al., 2010; Trousil et al., 2014). These findings arise from different models and measurement levels and do not establish one unified pathway. MSI can test whether membrane-lipid classes occupy the same pathologic compartments, but molecular identities require MS/MS. Protein colocalization generates a pathway hypothesis and does not replace measurement of the lipid or its flux.

The third link is fatty-acid fate across energy, storage, and inflammation. ACSL1-associated FAO has been linked to proliferation and metastasis. Pilot measurements of sterol O-acyltransferase 1 and cholesteryl esters nominate neutral-lipid storage. In PTEN-deficient models, omega-3 polyunsaturated fatty acids modulate COX-2/PGE2, whereas glycolytic inhibition reduces lipogenesis and tumor growth, linking glucose carbon to synthesis (Ayyagari et al., 2023; Byrne et al., 2014; Pan et al., 2015; Zhou et al., 2024). These branches may occur in different cells and regions and may change with molecular subtype and nutrient state. Spatial lipidomics should first localize oxidative, cholesterol-esterification, and PGE2-related compartments. Tracing and perturbation must then determine which branch sustains the tumor.

#### Direct spatial evidence and near-term priorities

The strongest direct patient evidence is a DESI-MSI study of 50 cancers and 14 benign tissues. Phosphatidic acid (PA)-, phosphatidylethanolamine (PE)-, phosphatidylserine (PS)-, and phosphatidylinositol (PI)-related signals were enriched in malignant regions and supported internal tissue classification (Paraskevaidi et al., 2025). This study anchored membrane-lipid differences to pathology rather than to serum or a tissue homogenate. PA enrichment does not establish phospholipase D flux, and the study did not show generalization across histotypes or centers or demonstrate clinical net benefit. Serum ultra-high-performance LC-MS/MS candidates and circulating palmitoleate or arachidonate provide non-spatial comparisons but cannot identify a tissue source (Cheng et al., 2023; Yamazaki et al., 2026).

Supporting spatial studies can help interpret direct lipid maps, but their evidentiary boundaries must remain explicit. Monosaccharide MALDI-MSI identified metabolic differences among tumor, peritumoral, and adjacent regions. Plasma and serum metabolomics nominated obesity-related detection and recurrence candidates (Audet-Delage et al., 2018; Njoku et al., 2021; Xia et al., 2025). Spatial cellular ecology, single-cell or spatial transcriptomics, HER2 heterogeneity, and protein imaging associated with lymph-node metastasis provide coordinates for vessels, malignant-cell states, and metastatic interfaces (Chui et al., 2026; Flesken-Nikitin et al., 2025; Mittal et al., 2016; Wu et al., 2025). These data can guide sampling and colocalization. Monosaccharides, transcripts, and proteins are not direct spatial lipid evidence and cannot independently establish a lipid’s cellular source.

The next stage should replicate direct phospholipid signals within molecular subtypes and place candidate nodes into a pathology-origin-flux-function chain. Lysophosphatidylcholine acyltransferase 1 changes phospholipid and triacylglycerol composition and promotes stemness and metastasis. Squalene epoxidase links cholesterol synthesis to PI3K-AKT signaling, whereas the RNA-binding motif protein 3-SLC7A11 axis suggests ferroptosis vulnerability (Ma L. et al., 2023; Wang et al., 2023; Zhao et al., 2022). These mechanisms merit direct testing in malignant glands, myometrial invasive fronts, and residual lesions. Current preclinical evidence cannot determine fertility-sparing treatment, adjuvant therapy, or immunotherapy. A defensible near-term objective is to show added recurrence or response information beyond POLE/MMR/p53/NSMP classification and standard pathology.

### Cervical cancer: HPV-driven membrane and inflammatory remodeling

#### From persistent infection to invasion and treatment resistance

Cervical cancer has the clearest etiologic trajectory. Persistent high-risk HPV infection is a necessary cause, and failed viral clearance can be followed by precancer, local invasion, and metastasis (American Cancer Society, 2026a; Bray et al., 2024; Crosbie et al., 2013; World Cancer Research Fund, 2026a; World Health Organization, 2021; World Health Organization, 2026). HPV vaccination and HPV-based screening reduce the risk of invasive cancer or precancer, yet global burden remains shaped by unequal access to vaccination, screening, follow-up, pathology, and treatment (European Society of Gynaecological Oncology, 2026; Falcaro et al., 2021; Lei et al., 2020; Ronco et al., 2014; World Health Organization, 2021). Spatial lipidomics should not bypass established prevention by seeking a replacement population-screening test. It should instead identify which persistent lesions progress, which invasive fronts form metastatic niches, and which regions remain after chemoradiotherapy.

Treatment is guided by stage, imaging, and pathology. Early disease is treated mainly with surgery or definitive radiotherapy, locally advanced disease with cisplatin-based chemoradiotherapy, and recurrent or metastatic disease with antiangiogenic agents, immune-checkpoint inhibitors, or antibody-drug conjugates (Cibula et al., 2023; Coleman et al., 2021; Colombo et al., 2021; Fischerova et al., 2024; Lorusso et al., 2024; McCormack et al., 2024; Mileshkin et al., 2023; Oaknin et al., 2024; Rose et al., 1999; Tewari et al., 2014; Tewari et al., 2022; Vergote et al., 2024). These trials define the comparators that a spatial candidate must outperform, but none validates a lipid biomarker. More appropriate designs should sample newly diagnosed locally advanced disease, post-treatment residual disease, and recurrent or metastatic disease separately. Lipid features should be modeled with stage, imaging, HPV background, and treatment exposure rather than merging different treatment settings into a generic resistance endpoint.

#### How HPV oncoproteins connect to lipid metabolism

HPV-associated lipid remodeling begins with sustained viral reprogramming of squamous epithelial differentiation and host signaling, not with proliferative demand alone. E6 and E7 disrupt tumor suppression and cell-cycle control. Stratified epithelial models further show that E6 or E6/E7 alters glycerophospholipids, sphingolipids, and oxidized lipids. HPV16 E6/E7 regulates COX-2 transcription, and E5 induces the PGE2 receptor EP4 (Crosbie et al., 2013; Gore et al., 2025; Oh et al., 2009; Spurgeon et al., 2026; Subbaramaiah and Dannenberg, 2007). These observations define a testable etiologic chain. Persistent viral expression changes membrane production and inflammatory lipid signaling, abnormal epithelium adapts to oxidative and immune selection, and distinct lipid states emerge at progression and invasion interfaces. Human-tissue validation by HPV type and lesion stage remains necessary, and cell models cannot establish one spatial state across all patients.

Studies of lymph-node metastasis further nominate a synthesis-export-uptake-invasion network. FASN promotes metastasis through cholesterol remodeling and lymphangiogenesis, and circZFR/YTHDF3 regulates its translation. Oleate in tumor-derived exosomes promotes lymphangiogenesis through AKT-mechanistic target of rapamycin signaling. Differences in fatty-acid transporters between precursor and malignant lesions, together with CD36-TGF-beta cooperation, connect exogenous uptake with epithelial-mesenchymal transition (An et al., 2022; Deng et al., 2019; Du et al., 2022; Li J. et al., 2026; Zhou et al., 2025). These studies suggest that tumor cells, exosomes, lymphatics, and stroma jointly shape a premetastatic niche. They do not show that every component acts in one patient or in a fixed direction. Spatial validation should pair tumor center, invasive front, and lymph node and identify lipid source and destination separately.

Ferroptosis connects membrane-lipid substrates, oxidative stress, and cisplatin response. FASN-SLC7A11, the mitochondrial dicarboxylate carrier SLC25A10, Krueppel-like factor 14-GPX4, and a peptide encoded by an HPV E6/E7-induced long non-coding RNA regulate ferroptosis through lipid supply, antioxidant defense, or peroxide clearance. Erucic acid derived from *Lactobacillus crispatus* induces PPAR-delta-dependent ferroptosis in organoids, suggesting a local microbiome-fatty-acid interface (Ma et al., 2025; Qi et al., 2025; Wang et al., 2025; Ye et al., 2024; Zhen et al., 2025). Patient associations with PGE2 receptor EP3, HPV16-related redox proteomics, and multi-omic differences among cell lines with different HPV backgrounds further indicate inflammatory and oxidative heterogeneity (De Marco et al., 2012; Heidegger et al., 2017; Liu et al., 2023). These preclinical findings generate radiosensitization or chemosensitization hypotheses. A single ferroptosis score cannot replace validation of specific substrates, cellular sources, and a therapeutic safety window.

#### Direct spatial evidence and near-term priorities

Direct spatial lipid evidence in cervical cancer remains sparse. Xu et al. combined tissue MSI with spatial transcriptomic information in two tumors, mapping oleate and observing overlap with receptor for activated C kinase 1 (RACK1), acetyl-CoA carboxylase 1 (ACC1), and FASN. Complementary perturbation and rescue supported a RACK1-AKT/mechanistic target of rapamycin-SREBP1 lipogenic pathway (Xu et al., 2025). The study created a cross-scale hypothesis connecting oleate-associated regions, synthetic control, and invasion, consistent with non-spatial oleate-CD36-Src/ERK evidence (Yang et al., 2018). The two patients were not fully concordant. Colocalization cannot distinguish intracellular synthesis, exogenous uptake, or intercellular exchange, and it does not support dietary causality or clinical biomarker utility.

A second study used atmospheric-pressure matrix-assisted laser desorption/ionization MSI (AP-MALDI-MSI) to compare tumors from six patients with or without lymph-node metastasis. Several putative phosphatidylcholine (PC) and PE ions differed between groups (Zhang et al., 2026). This finding provides a direct but very early tissue anchor for nodal risk. Each group contained only three patients, structural confirmation was incomplete, and no external validation was performed. Spatial fatty-acid degradation at the tumor periphery, early serum lipid candidates, bulk-tissue metabolites, and longitudinal changes during chemoradiotherapy provide process, liquid, or longitudinal evidence. None is interchangeable with direct spatial lipid measurement (Abudula et al., 2020; Cheng et al., 2020; Choi et al., 2024; Ge et al., 2026; Lin et al., 2026).

The most informative near-term settings are lymph-node metastasis, the invasive-front or residual ecosystem, and chemoradiotherapy response. Prospective cohorts should expand paired sampling and confirm PC, PE, oleate, and related candidates by MS/MS or orthogonal lipidomics. Pathologic compartment, HPV status, cellular origin, and isotope tracing should then be integrated before patient-level comparison with imaging, stage, and outcome. Current evidence supports mechanism discovery and selection of sampling regions. It does not support population screening, routine staging, treatment selection, or recurrence monitoring.

Table 1 compares the direct spatial lipid studies and the highest defensible inference supported by each study.

**TABLE 1:** Direct spatial lipid evidence in ovarian, endometrial, and cervical cancers.

Cancer and study	Sample/model and platform	Direct spatial lipid finding	Defensible near-term use	Critical boundary
Ovarian, Dória et al. (2016)	Patient ovarian tissue; DESI-MSI	Glycerophospholipid and related ion combinations separated normal, borderline, and epithelial tumors	Candidate pathologic-region recognition and histologic classification	Internal classification; incomplete identity for some ions; no external clinical-utility validation
Ovarian, Sans et al. (2017)	Patient serous ovarian tissue; ambient-ionization MSI	Fatty-acid-, ceramide-, and glycerophospholipid-related patterns tracked invasive phenotype	Candidate aggressiveness stratification	Patient holdout is not independent multicenter validation; no prospective decision utility
Ovarian, Liu et al. (2010)	Patient tissue; MSI, *in situ* MS/MS, and transcript integration	Sulfatide elevation localized to malignant epithelium	Tissue anchor for glycosphingolipid remodeling	Limited cohort; enzyme-transcript support does not establish lipid flux or treatment utility
Ovarian, Mukherjee et al. (2023)	Patient spatial subset (n = 3) with models; MALDI-MSI, tracing, and perturbation	PC was higher near adipocytes and fell with distance, whereas PI showed the reverse trend; supported G3P-glycerolipid coupling	Localization of the omental adipose niche and metastatic mechanism	Modules were not closed in the same patient; some ions were putative; no patient-level utility
Ovarian, shared genetic-model lineage (2016–2024) (Paine et al., 2016; Sah et al., 2022; Ma X. et al., 2023)	Dicer/Pten-associated HGSOC mouse lineage; whole-reproductive-system and ultrahigh-resolution MSI	Organ- and stage-dependent glycerophospholipid, sphingolipid, and ether-lipid distributions	Early-evolution hypotheses and candidate prioritization	Three reports are not three independent patient replications; animal findings cannot define a screening biomarker
Ovarian, Nguyen et al. (2024)	Ovarian-tumor proof of concept; lithium-doped nano-DESI-MSI	Broader low-abundance lipid coverage and improved PGD2/PGE2-related localization	Method development for inflammatory-lipid and isomer localization	Coverage does not establish complete structure, causality, or patient use
Ovarian, Gorman et al. (2024)	Ferroptosis-treated ovarian-tumor xenograft; multimodal MALDI/SIMS	Intervention-responsive regions differed in iron and lipid composition	Discovery of spatial pharmacodynamic and ferroptosis-sensitive regions	Model endpoints do not prove patient efficacy, direct drug targeting, or clinical prediction
Ovarian, Cumin et al. (2022)	Ovarian cancer tissue/models; spatial glycosphingolipidomics and genetic perturbation	Globosides favored tumor regions and gangliosides favored stromal regions and were linked to plasticity	Compartment-specific glycosphingolipid mechanism	Spatial distribution and model perturbation do not establish patient-level therapeutic or prognostic utility
Endometrial, Paraskevaidi et al. (2025)	64 women (50 cancers and 14 benign samples); DESI-MSI	PA-, PE-, PS-, and PI-related signals were enriched in cancer and supported internal classification	Benign-malignant tissue recognition and localization of membrane remodeling	Single-cohort internal classification; no cross-histotype, cross-center, or net-benefit validation
Cervical, Zhang et al. (2026)	Spatial subset n = 6 (3 node-positive and 3 node-negative); AP-MALDI-MSI, 17 μm	Five putative PC and five putative PE ions showed higher signals in the metastatic group	Candidate lymph-node-risk discovery	Extremely small exploratory cohort; incomplete structure; no external or patient-level validation
Cervical, Xu et al. (2025)	Spatial profiles n = 2; tissue MSI and spatial transcriptomics	Oleate overlapped RACK1, ACC1, and FASN; perturbation supported a lipogenic axis	Localization of a candidate local lipogenic mechanism	Patient inconsistency; colocalization does not prove source or flux; no prognostic, predictive, or clinical utility

Direct spatial lipid evidence requires a lipid molecule or lipid-annotated ion to be measured while tissue coordinates are retained. Paine et al. (2016), Sah et al. (2022), and Ma et al. (2025) share a model lineage and are grouped accordingly. HGSOC, high-grade serous ovarian carcinoma; MSI, mass spectrometry imaging; PA, phosphatidic acid; PC, phosphatidylcholine; PE, phosphatidylethanolamine; PI, phosphatidylinositol; PS, phosphatidylserine.

#### Cross-cancer integration: shared functional axes and non-interchangeable etiologic ecologies

The three cancers do not share one fixed lipid fingerprint. They share functional demands for lipid supply, membrane homeostasis, energy, and redox control. Ovarian cancer is selected by peritoneal dissemination and the lipid-rich omentum. Endometrial cancer develops where obesity, estrogen, and insulin signaling converge with PI3K abnormalities. Cervical cancer begins with HPV-oncoprotein remodeling of squamous epithelium and inflammatory signaling (Bokhman, 1983; Nieman et al., 2011; Spurgeon et al., 2026). Cross-cancer comparisons can therefore ask whether uptake, synthesis, desaturation, FAO, PGE2, or ferroptosis defense is required. They cannot assume the same donor cell, net direction, dominant compartment, or drug dependence.

Membrane remodeling is currently the most directly comparable spatial layer. Ovarian sulfatides and glycosphingolipids, endometrial PA/PE/PS/PI, and cervical PC/PE and oleate have tissue coordinates of varying strength (Liu et al., 2010; Paraskevaidi et al., 2025; Trousil et al., 2014; Xu et al., 2025; Zhang et al., 2026). The same m/z or a similar regional abundance can represent the same molecule only when identification level, pathologic compartment, platform, and batch are comparable. Regional signals are also influenced by cellular composition. Adipocyte proximity, vascular density, necrosis, and invasive fronts can each reshape a map. Spatial proximity generates a donor-recipient hypothesis but cannot determine the direction of transfer (Mukherjee et al., 2023; Wu et al., 2025; Zhou et al., 2024).

PGE2 and ferroptosis offer strong cross-cancer mechanistic interfaces, but a shared endpoint does not imply a shared upstream pathway. COX/PGE2-related inflammation is supported in all three cancers, yet cellular source and spatial distribution have not been established in a common framework (Brencicova et al., 2017; Pan et al., 2015; Subbaramaiah and Dannenberg, 2007). Ferroptosis depends on peroxidizable phospholipids and antioxidant defense. Ovarian evidence emphasizes SCD1/FADS2 and treatment-responsive regions, endometrial evidence includes RNA-binding motif protein 3/SLC7A11, and cervical evidence links FASN/SLC7A11 and GPX4 with cisplatin response (Gorman et al., 2024; Tesfay et al., 2019; Wang et al., 2023; Wang et al., 2025). Measurement and validation strategies can be shared, but doses, patient-selection criteria, and combinations cannot be transferred directly across diseases.

Spatial multi-omics should begin with the lipid. Candidate lipids and compartments should first be confirmed in tissue, after which spatial transcriptomic or proteomic data can narrow possible cellular sources. Microdissection, sorting, isotope tracing, and perturbation should then validate source, flux, and function. Beginning with a transcript or protein, inferring a lipid, and treating colocalization as metabolic flux converts proxy evidence into a false direct measurement. What can transfer across the three cancers is the evidence ladder from clinical question to molecular identity, cellular origin, flux, function, and patient validation, not an ion peak, threshold, or classifier (Dória et al., 2016; Ge et al., 2026; Lin et al., 2026; Mukherjee et al., 2023; Paraskevaidi et al., 2025; Pietkiewicz et al., 2022; Xu et al., 2025; Zhang et al., 2026).

Figure 4 visualizes the evidence-weighted links between spatial lipid states and plasticity, immunity, treatment response, and proposed clinical uses.

**FIGURE 4:**
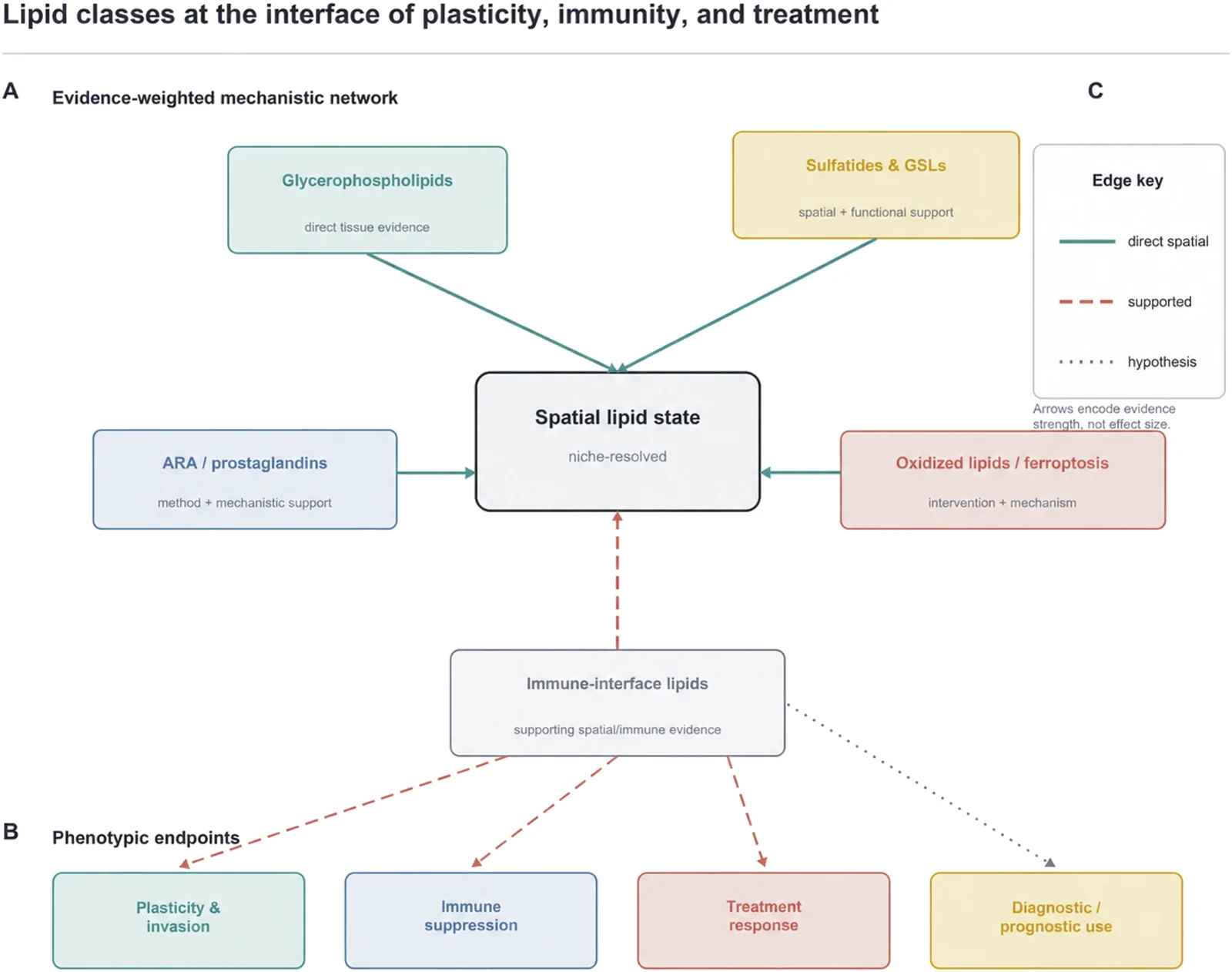
Lipid-mechanistic network and evidence depth across the three gynecologic cancers. Shared nodes include uptake, synthesis, desaturation, membrane remodeling, fatty-acid oxidation, PGE2, and ferroptosis. Upstream causes, dominant tissue compartments, and validation depth differ by disease (Brencicova et al., 2017; Dória et al., 2016; Li et al., 2012; Mukherjee et al., 2023; Nieman et al., 2011; Pan et al., 2015; Paraskevaidi et al., 2025; Spurgeon et al., 2026; Subbaramaiah and Dannenberg, 2007; Tesfay et al., 2019; Wang et al., 2023; Wang et al., 2025; Xu et al., 2025; Zhang et al., 2026). **(A)** Evidence-weighted mechanistic network connecting lipid classes with the spatial lipid state. **(B)** Phenotypic endpoints involving plasticity, immunity, treatment response, and diagnostic or prognostic use. **(C)** Edge key indicating direct spatial evidence, supported links, and hypotheses.

Table 2 aligns disease-specific clinical problems with lipid niches, evidence depth, and the next testable use.

**TABLE 2:** Disease-specific clinical problems, lipid niches, and spatial research priorities.

Cancer	Disease-specific clinical problem	Lipid niche and priority programs	Direct spatial evidence	Near-term testable use	Currently unsupported use
Ovarian	HGSOC often presents after peritoneal dissemination; screening has not reduced mortality in average-risk women; recurrence and resistance persist after maintenance therapy (Us Preventive Services Task Force, 2018; World Cancer Research Fund, 2026c)	Omental adipocyte supply; CD36/FABP4 uptake; G3P-glycerolipid synthesis; FASN/SCD1; lipid droplets-FAO; PGE2; ferroptosis	Deepest of the three, spanning patient-tissue classification, adipose niches, glycosphingolipid compartments, and intervention imaging (Dória et al., 2016; Mukherjee et al., 2023)	Validate recurrence- or response-associated features in primary, omental, and residual compartments against pathology, BRCA/homologous-recombination status, and standard variables	Population screening, routine maintenance selection, or recurrence monitoring
Endometrial	Obesity and metabolism shape disease burden; molecular classification informs risk; fertility preservation, adjuvant escalation, and advanced treatment require precise stratification (Berek et al., 2023; World Cancer Research Fund, 2026b)	*De novo* synthesis and desaturation in an obesity-hormone-PI3K context; choline-phospholipid/sphingolipid remodeling; FAO; cholesterol esterification	Concentrated in PA/PE/PS/PI remodeling in one 64-patient DESI-MSI cohort (Paraskevaidi et al., 2025)	Replicate by molecular subtype and pathologic compartment and test gain for myometrial invasion, recurrence, or treatment stratification	Replacement of POLE/MMR/p53/NSMP classification, fertility-sparing decisions, or prediction of immunotherapy benefit
Cervical	Vaccination and screening are effective but coverage gaps persist; nodal metastasis, chemoradiotherapy residual disease, and recurrence remain incompletely stratified (European Society of Gynaecological Oncology, 2026; World Cancer Research Fund, 2026a)	HPV E6/E7-driven membrane and COX/PGE remodeling; fatty-acid synthesis/uptake; exosomal oleate; oxidative stress; ferroptosis	Limited to an n = 6 PC/PE nodal comparison and n = 2 oleate-RACK1 colocalization (Xu et al., 2025; Zhang et al., 2026)	Expand node-status and tumor-edge cohorts and perform longitudinal response studies at defined treatment points	Population screening, replacement of HPV pathways, routine staging, or treatment selection

Near-term uses are next-stage validation tasks, not established clinical indications. FAO, fatty-acid oxidation; G3P, glycerol-3-phosphate; MMR, mismatch repair; NSMP, no specific molecular profile; other lipid abbreviations are defined in Table 1.

## Discussion: from spatial discovery to clinical utility

### From credible ions to interpretable mechanisms

The first translational rule is that a conclusion must not exceed its evidentiary level. A spatial map can answer where only after sampling and preparation are controlled and ion identity is supported by MS/MS or an orthogonal method. Pathologic segmentation, spatial multi-omics, and cell-specific validation are then required to address cellular origin. Stable-isotope tracing addresses metabolic direction, whereas genetic or pharmacologic perturbation and rescue address causality (Condina et al., 2019; Gorman et al., 2024; Mukherjee et al., 2023; Nguyen et al., 2024; Schwamborn, 2017; Thomas and Chaurand, 2014; Wu et al., 2025; Xia et al., 2025; Xu et al., 2025). Ovarian adipocyte-tumor interaction, endometrial phospholipid imaging, and cervical oleate-RACK1 colocalization represent different levels of maturity. The first begins to connect space, flux, and function; the latter two remain largely at regional localization or source hypothesis (Mukherjee et al., 2023; Paraskevaidi et al., 2025; Xu et al., 2025).

### From patient-level models to clinical decisions

A mechanistic finding does not automatically create a clinical test. Candidate features must be split into training, tuning, and testing sets by patient rather than by pixel. Peak processing, normalization, features, and thresholds should be locked before external validation. Studies should report discrimination, calibration, failure rates, and confidence intervals in the intended population (Dória et al., 2016; Ledoux et al., 2026; Sans et al., 2017; Schwamborn, 2017). Diagnostic models should be compared with standard pathology, prognostic models should test gain beyond stage and molecular classification, and predictive models must demonstrate a biomarker-treatment interaction. A circulating lipid may be a detection candidate without arising from a tumor compartment. A spatially associated lipid may be a mechanistic readout without being a druggable driver (Cheng et al., 2020; Cheng et al., 2023; Sutphen et al., 2004).

### Disease-specific paths and implementation

Each cancer must repair a different weakest link. Ovarian cancer requires multicenter patient-level replication that connects primary tumors, omental metastases, and residual disease. Endometrial cancer requires independent replication of PA/PE/PS/PI signals within molecular subtypes. Cervical cancer requires larger paired cohorts by nodal status and invasive front, together with structural confirmation of PC, PE, and oleate (Dória et al., 2016; Paraskevaidi et al., 2025; Xu et al., 2025; Zhang et al., 2026). Study design should also incorporate consent for sample use, pathology interfaces, data governance, turnaround time, cost, and resource accessibility. High accuracy at one center is not implementation, and case-control separation does not show fewer missed diagnoses, less overtreatment, or lower mortality (Abacjew-Chmylko et al., 2026; Cibula et al., 2023; Concin et al., 2025; Leung et al., 2024; Menon et al., 2021; World Health Organization, 2020).

Figure 5 places discovery, external validation, and prospective utility on a single gated translational pathway.

**FIGURE 5:**
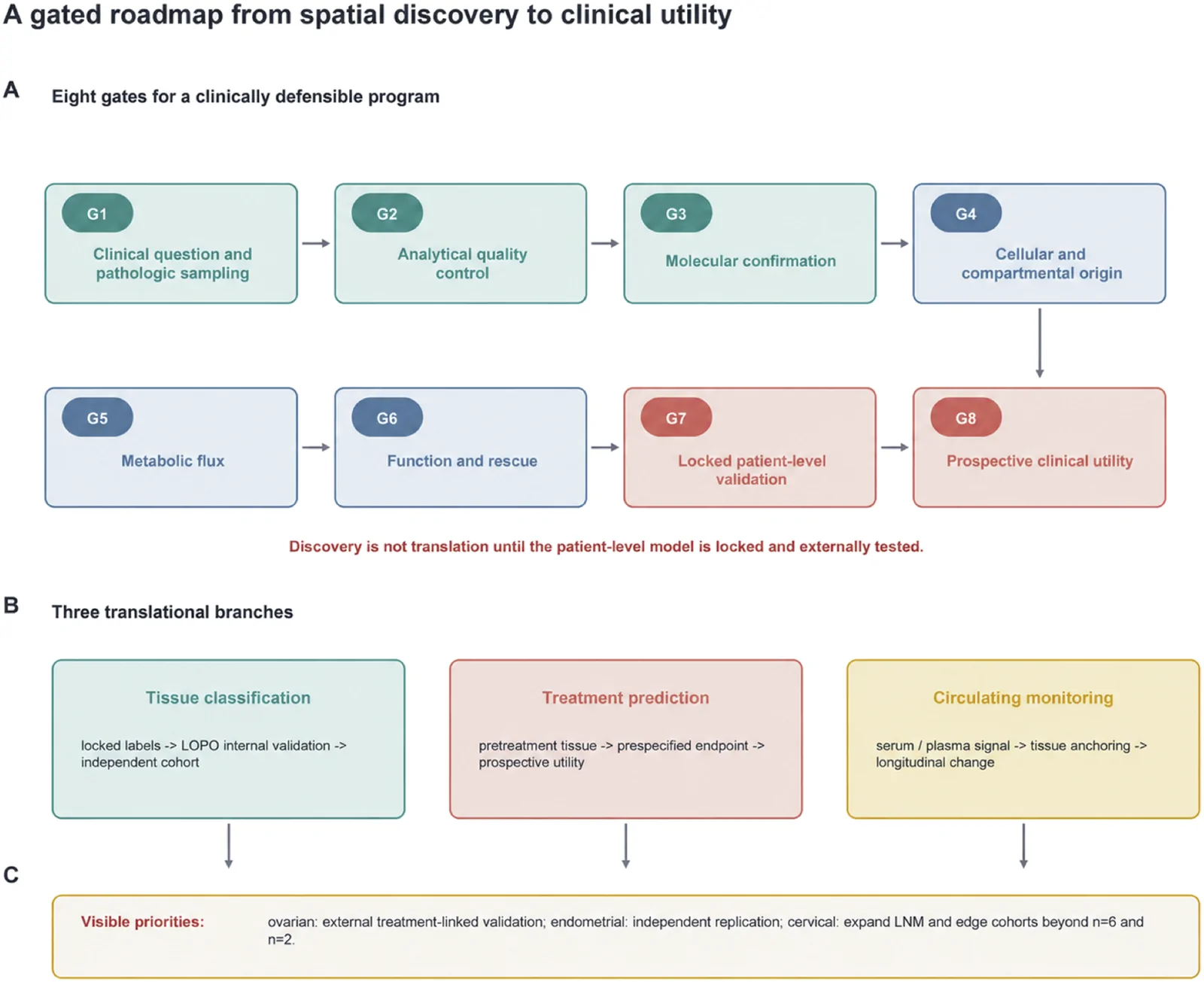
Clinical translation pathway for spatial lipidomics. Candidate discovery is followed by analytical control, molecular identification, cellular-source assignment, metabolic flux, functional perturbation and rescue, patient-level external validation, and assessment of clinical decision gain (Condina et al., 2019; Dória et al., 2016; Ge et al., 2026; Gorman et al., 2024; Ledoux et al., 2026; Leung et al., 2024; Lin et al., 2026; Mondal et al., 2019; Mukherjee et al., 2023; Nguyen et al., 2024; Paraskevaidi et al., 2025; Pietkiewicz et al., 2022; Schwamborn, 2017; Thomas and Chaurand, 2014; Wu et al., 2025; Xia et al., 2025; Xu et al., 2025; Zhang et al., 2026). **(A)** Eight validation gates from pathologic sampling to prospective clinical utility. **(B)** Translational branches for tissue classification, treatment prediction, and circulating monitoring. **(C)** Disease-specific validation priorities.

Table 3 operationalizes the progression from spatial discovery to clinical utility as eight validation gates.

**TABLE 3:** Eight validation gates from spatial discovery to clinical utility.

Gate	Minimum methodological requirement	Defensible statement after passing	Claim that remains unsupported
G1 Clinical question and pathologic sampling	Prespecify the population, decision point, and comparator; pathologically define tumor, stroma, necrosis, adipose tissue, and invasive front; record complete sampling and preparation metadata (Schwamborn, 2017; Thomas and Chaurand, 2014)	The candidate region matches the clinical question	Diagnostic, prognostic, or predictive utility
G2 Analytical quality control	External process control, randomized run order, replicate sections, drift and ion-suppression assessment; patient as statistical unit (Condina et al., 2019)	The workflow is reproducible under specified conditions	Biological mechanism or cross-center transportability
G3 Molecular confirmation	Accurate mass, *in situ* MS/MS, orthogonal LC-MS/MS or ion mobility, and standards or isomer-specific methods when required (Nguyen et al., 2024; Xia et al., 2025)	The lipid can be named at the declared confidence level	A single m/z establishes double-bond position, stereochemistry, or absolute concentration
G4 Cellular and compartmental origin	Pathologic segmentation plus spatial transcript or protein data, microdissection, cell sorting, or cell-specific validation; registration and neighborhood sensitivity assessed (Mukherjee et al., 2023; Wu et al., 2025; Xu et al., 2025)	A lipid is consistent with a specified cellular or compartmental source	Donor-recipient direction or intercellular transfer
G5 Metabolic flux	Stable-isotope tracing separates synthesis, uptake, storage, and degradation and maps labeled substrate fate to tissue regions (Mukherjee et al., 2023)	Regional metabolic direction receives direct support	Static abundance itself is flux or causality
G6 Function and rescue	Genetic or pharmacologic perturbation, time and dose relationships, target specificity, functional rescue, and reproduction in a structure-preserving model (Gorman et al., 2024; Xu et al., 2025)	The node is functional in the specified model and endpoint	Model sensitization equals patient benefit or an acceptable therapeutic window
G7 Locked patient-level validation	Patient-level training, tuning, and testing; preprocessing and thresholds locked before external testing; independent multicenter discrimination, calibration, and failure rates (Dória et al., 2016; Ledoux et al., 2026)	The locked feature is clinically valid in the intended population outside the development center	Internal cross-validation establishes generalizability or implementation
G8 Prospective clinical utility	Direct comparison with standard pathology, molecular models, or treatment; prespecified endpoint; decision net benefit, turnaround, cost, access, and governance assessed	The test improves management at a specified decision point	Utility for one use transfers to screening, prognosis, treatment prediction, or monitoring

The gates are progressive, although an early discovery study need not complete all eight. The governing rule is that a claim must not exceed the highest gate actually passed.

## Conclusion

Lipid remodeling in gynecologic malignancies is jointly selected by etiology and tissue ecology. Ovarian cancer strengthens exogenous uptake, glycerolipid synthesis, and post-treatment energy adaptation during peritoneal dissemination and omental colonization. Endometrial cancer connects obesity, estrogen, and insulin with PI3K-SREBP1-driven membrane production. Cervical cancer links HPV oncoproteins with membrane lipids, PGE2 signaling, and oxidative stress. Uptake, synthesis, desaturation, FAO, membrane remodeling, PGE2, and ferroptosis can operate in all three diseases, but these shared functions do not erase disease-specific drivers and compartments.

The central contribution of spatial lipidomics is to return bulk lipid differences to malignant epithelium, adipose tissue, stroma, vessels, necrosis, and invasive fronts. Evidence remains markedly asymmetric. Ovarian cancer includes patient-tissue classification, omental-ecology studies, and intervention imaging. Endometrial evidence centers on one 64-patient phospholipid-imaging cohort. Cervical evidence consists mainly of an n = 6 PC/PE study and an n = 2 oleate analysis (Dória et al., 2016; Mukherjee et al., 2023; Paraskevaidi et al., 2025; Xu et al., 2025; Zhang et al., 2026). These studies establish spatial heterogeneity and generate mechanisms, but they do not yet establish a routine clinical assay.

Progress now depends less on adding unconfirmed peaks than on closing the chain from clinical question and analytical control to molecular identity, cellular origin, flux, rescue, external validation, and decision gain. The most attainable near-term value is localization of disease-specific niches, guidance of mechanistic experiments, and prioritization of patient subsets for prospective study. No candidate lipid peak should guide screening, diagnosis, treatment selection, or surveillance before multicenter replication, comparison with standard models, and implementation utility are demonstrated.
